# Web-Based Intervention for Physical Activity and Fruit and Vegetable Intake Among Chinese University Students: A Randomized Controlled Trial

**DOI:** 10.2196/jmir.7152

**Published:** 2017-04-10

**Authors:** Yan Ping Duan, Julian Wienert, Chun Hu, Gang Yan Si, Sonia Lippke

**Affiliations:** ^1^ Department of Physical Education Faculty of Social Sciences Hong Kong Baptist University Hong Kong China (Hong Kong); ^2^ Institute of Social Medicine and Epidemiology University of Lübeck Lübeck Germany; ^3^ Department of Health & Physical Education The Education University of Hong Kong Hong Kong China (Hong Kong); ^4^ Bremen International Graduate School of Social Sciences Jacobs University Bremen Bremen Germany; ^5^ Department of Psychology & Methods Jacobs University Bremen Bremen Germany

**Keywords:** Web-based intervention, physical activity, fruit and vegetable intake, university students, motivational indicators, volitional indicators

## Abstract

**Background:**

Ample evidence demonstrates that university students are at high risk for sedentary behaviors and inadequate fruit and vegetable intake (FVI). Internet-based interventions for multiple health behavior appear to be promising in changing such unhealthy habits. Limited randomized controlled trials have tested this assumption among Chinese university students.

**Objective:**

Our objective was to test the efficacy of an 8-week Web-based intervention compared with a control group condition to improve physical activity (PA) and FVI in Chinese university students. The intervention content was based on the health action process approach, and developed on the basis of previous evidence from the Western hemisphere. We evaluated self-reported data including PA and FVI, stages of change for PA and FVI, and motivational (risk perception, outcome expectancies, self-efficacy), volitional (action planning, coping planning, social support), and distal (intention, habit) indicators for PA and FVI, as well as perceived mental health outcomes (quality of life, depression).

**Methods:**

In a randomized controlled trial, we recruited 566 university students from one university in the central region of China during their general physical education class. After random allocation and exclusion of unsuitable participants, we assigned 493 students to 1 of 2 groups: (1) intervention group: first 4 weeks on PA and subsequent 4 weeks on FVI, (2) control group. We conducted 3 Web-based assessments: at the beginning of the intervention (T1, n=493), at the end of the 8-week intervention (T2, n=337), and at a 1-month follow-up after the intervention (T3, n=142). The entire study was conducted throughout the fall semester of 2015.

**Results:**

Significant time ⨯ group interactions revealed superior intervention effects on FVI; motivational, volitional, and distal indicators of FVI; and PA behavior changes, with an effect size (η^2^) ranging from .08 to .20. In addition, the overall intervention effects were significant for stage progression to the action group from T1 to T2 in PA (χ^2^_1_=11.75, *P*=.001) and FVI (χ^2^_1_=15.64, *P*=.03). Furthermore, the intervention effect was seen in the improvement of quality of life (*F*_3,492_=1.23, η^2^=.03, *P*=.02).

**Conclusions:**

This study provides evidence for the efficacy of a Web-based multiple health behavior intervention among Chinese university students tested with different outcome variables. Future research should address the high dropout rate and optimize the most effective components of this intervention.

**Trial Registration:**

Clinicaltrials.gov NCT01909349; https://clinicaltrials.gov/ct2/show/NCT01909349 (Archived by WebCite at http://www.webcitation.org/6pHV1A0G1)

## Introduction

Being physically active and eating healthily are known to reduce the risk of developing noncommunicable diseases such as cancer, cardiovascular disease, obesity, and type 2 diabetes [1]. Few young people, however, reach the recommendations of health behavior for the prevention of noncommunicable diseases. Considerable evidence indicates that more than half of university students do not achieve at least 150 accumulated minutes of moderate physical activity (PA) per week [2,3]. Additionally, some studies show that university students and young adults aged 18-24 years consume fewer than the recommended 5 daily servings of fruits and vegetables [4,5].

University students are in the transition stage from late adolescence to adulthood. Unhealthy lifestyle habits, which are reinforced during this phase, often persist into later life and lead to long-term negative health outcomes [6]. Strategies to foster a healthy lifestyle in university students are therefore essential. Some evidence reveals that multiple behavior health interventions can promote both exercise and healthy diets in university students [1,7,8]. Thus, behavioral interventions for university students are required to support long-lasting behavior changes beyond late adulthood. Most of these studies, however, have been conducted only in Western societies.

Using Internet technology to promote health behavior change has several advantages [9]. In particular, an Internet intervention can (1) be delivered to large numbers of people at a low cost, (2) ensure that the intervention is accessible at any time and any location, and (3) provide follow-ups and feedback with personalized and tailored methods. In addition, the use of Internet technology is particularly relevant to young adults, who are the major users of such technology [10]. Moreover, tailored Web-based health behavior interventions that try to address specific characteristics of participants by providing information and feedback based on previously provided information (eg, assessed via questionnaire) harness the potential to provide a more user-specific experience with higher relevance for each participant [11,12]. A meta-analysis by Lustria et al [12] concluded that tailored Web-based interventions could lead to significant improvement in health outcomes at posttesting (*d*=.14) and follow-up (*d*=.16). Such tailored Web-based behavioral interventions, however, have very rarely been systematically evaluated in China. To the best of our knowledge, this study is the first test of a Web-based multiple behavior health intervention delivered to Chinese university students.

### The Health Action Process Approach

The theoretical backdrop of this study is the health action process approach (HAPA; [13]), which divides the health behavior change process into 2 phases. First is the motivational phase, in which people who do not intend to change their behavior (nonintenders) are motivated to develop their intentions. Afterward, they enter the second, or volitional, phase, in which people initiate and perform the behavior. Within the volitional phase, a distinction can be made between people who have the intention to perform a specific behavior but do not act (intenders) and people who already perform the behavior (actors). These distinctions allow for the interventions to address those variables that are relevant to specific processes in the sequential order. For example, before people can change unhealthy habits, they must become motivated to do so. Thus, such individuals may benefit most from interventions that increase risk perception, self-efficacy, and the promotion of positive outcome expectancies [13,14]. The idea is to lead the individual toward an explicit intention, by increasing their awareness of potential risks, highlighting the positive effects of a healthy lifestyle and addressing incorrect beliefs about negative effects. Upon forming the intention, people enter the volitional process. Individuals here benefit most from action and coping planning interventions [15], as such interventions help them translate their plans into behavior. Once people start to perform a healthy behavior, self-regulatory skills are most relevant for their maintenance progress. The behavior is mainly directed by self-efficacy, which regulates how effort is invested and how persistence is managed if barriers and setbacks occur. In addition, promoting perceived social support from the individuals’ social environment is equally important in preventing relapse [13].

### Study Objective

On the basis of this theory, previous interventions have been developed and conducted with people interested in reducing their cardiovascular risk in the Western hemisphere, where the intervention effects have been well supported [16,17]. This study aimed at testing the effectiveness of such an intervention for Chinese students. Our objective was to test the efficacy of an 8-week Web-based intervention (first 4 weeks on PA, and subsequent 4 weeks on fruit and vegetable intake [FVI]) compared with a control group condition to improve PA and FVI in Chinese university students. We examined the effects on behaviors, stages of change movement for PA and FVI, social-cognitive indicators for PA and FVI (motivational, volitional, and distal), and perceived mental health outcomes (quality of life and depression level).

We hypothesized that the main intervention effects would be (1) more behavioral change in PA and FVI (hypothesis 1a) and more stage progression to the action stage for PA and FVI (hypothesis 1b), (2) more improvements in motivational (risk perception, outcome expectancies, self-efficacy), volitional (action planning, coping planning, social support), and distal (intention, habit) indicators of behavior change (hypothesis 2), and (3) an increase in positive mental health outcomes, including higher quality of life and lower depression levels (hypothesis 3).

## Methods

### Participants and Procedure

**Figure 1:**
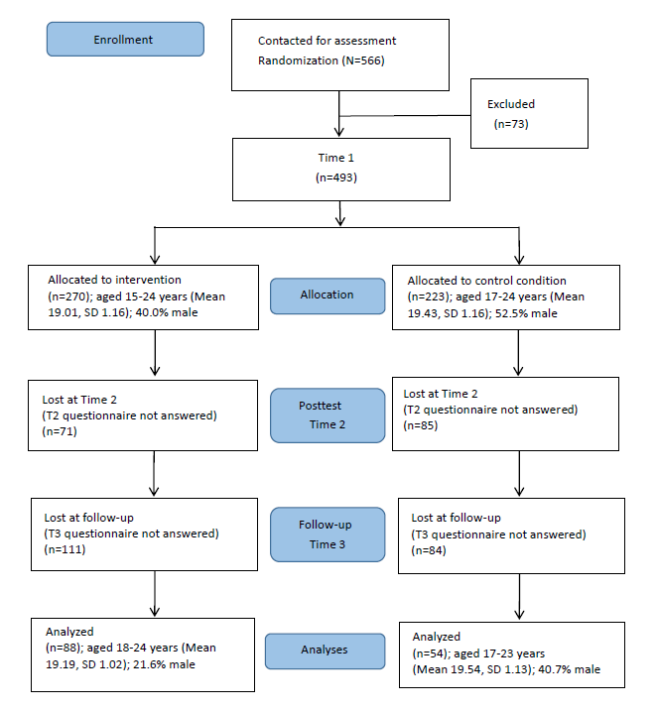
Flowchart of participant progress throughout the study phases.

Study participants were undergraduate students from one university in the central region of China. We contacted a total of 566 students in their first general physical education classes with the assistance of physical education lecturers during the fall semester of 2015. Of these, we excluded 73 (12.9%) after random allocation, including those who were collegiate athletes, had restrictions in terms of PA or FVI, or because they declined to participate. Subsequently, 493 (87.1%) students completed the online registration and provided personal information within 1 week, including 270 students in the intervention group (54.8%) and 223 students in the control group (45.2%). During the week after registration, we invited students in both groups to answer the first onscreen questionnaires (T1). Students in the intervention group were encouraged to participate in a Web-based program once a week during the following 8 weeks, while students in the control group did not receive any support. Upon completion of the 8-week intervention, students in both groups were invited to fill in the second onscreen questionnaire (T2), followed by the third onscreen questionnaire administration 1 month (T3) after the intervention. By the end of the 8-week intervention, 337 (68.4%) students were still participating, with 199 (59.1%) in the intervention group and 138 (41.0%) in the control group. The final longitudinal sample consisted of 142 students (28.8%), including 88 (62.0%) in the intervention group and 54 (38.0%) in the control condition (Figure 1). All website links for the questionnaire surveys at T1, T2, and T3, as well as for the weekly intervention program, were delivered via email. To boost the engagement of students, we sent text message reminders and physical education lecturers verbally reminded participants during class during the weekly intervention process and 3 measurement points. Moreover, students were offered an additional 5 marks to their final physical education examination score as an incentive in exchange for their participation at all 3 data collection waves.

All students were informed about the purpose of the study with an informed consent form. The study procedure was approved by the Committee for the Use of Human & Animal Subjects in Teaching & Research of Hong Kong Baptist University, as well as the Deutsche Gesellschaft für Psychologie in Germany (EK-A-SL022013), and was registered with ClinicalTrials.gov (NCT01909349; Multimedia Appendix 1 [18]).

### Intervention

The intervention comprised an 8-week Web-based intervention targeting social-cognitive indicators for health behavior change for PA and FVI, based on the HAPA model [13]. The intervention applied behavior change techniques such as providing information about behavioral risks and the benefits of behavior change, motivating the formation of intentions, prompting identification of barriers, prompting setting of specific goals, reviewing behavioral goals, providing feedback on performance, prompting practice and presenting follow-up prompts, motivating the planning of social support, and prompting relapse prevention [19]. Weeks 1-4 aimed at PA and weeks 5-8 aimed at FVI, as PA might act as a gateway behavior [20,21] and PA modules are the most favored ones in tailored eHealth lifestyle promotion [22]. Participants received 1 session per week, which lasted about 20 minutes.

The first session for each behavior targeted risk perception, outcome expectancies, and goal setting, and the second session targeted the development of action plans. The third session targeted the revision and adjustment of previously developed action plans, as well as the development of coping plans, while the fourth session targeted the revision and adjustment of previously developed coping plans and social support (Table 1).

**Table 1:** Intervention content and techniques for each week.

Session content		Physical activity				Fruit and vegetable intake			
		Week 1	Week 2	Week 3	Week 4	Week 5	Week 6	Week 7	Week 8
**Session 1**									
	Risk perception	Yes				Yes			
	Outcome expectancies	Yes				Yes			
	Goal setting	Yes				Yes			
**Session 2**									
	Development of action plans		Yes				Yes		
**Session 3**									
	Revision of action plans			Yes				Yes	
	Development of coping plans			Yes				Yes	
**Session 4**									
	Revision of coping plans				Yes				Yes
	Social support				Yes				Yes
Behavior change techniques [19]		Information on behavioral risks and benefits of behavior change, motivating intention formation, prompting specific goal setting	Feedback on performance, reviewing behavioral goals, prompting practice	Feedback on performance, presenting follow-up prompts, prompting relapse prevention, prompting barrier identification	Feedback on performance, prompting relapse prevention, motivating the planning of social support	Information behavioral risks and benefits of behavior change, motivating intention formation, prompting specific goal setting	Feedback on performance, reviewing behavioral goals, prompting practice	Feedback on performance, presenting follow-up prompts, prompting relapse prevention, prompting barrier identification	Feedback on performance, prompting relapse prevention, motivating the planning of social support

**Figure 2:**
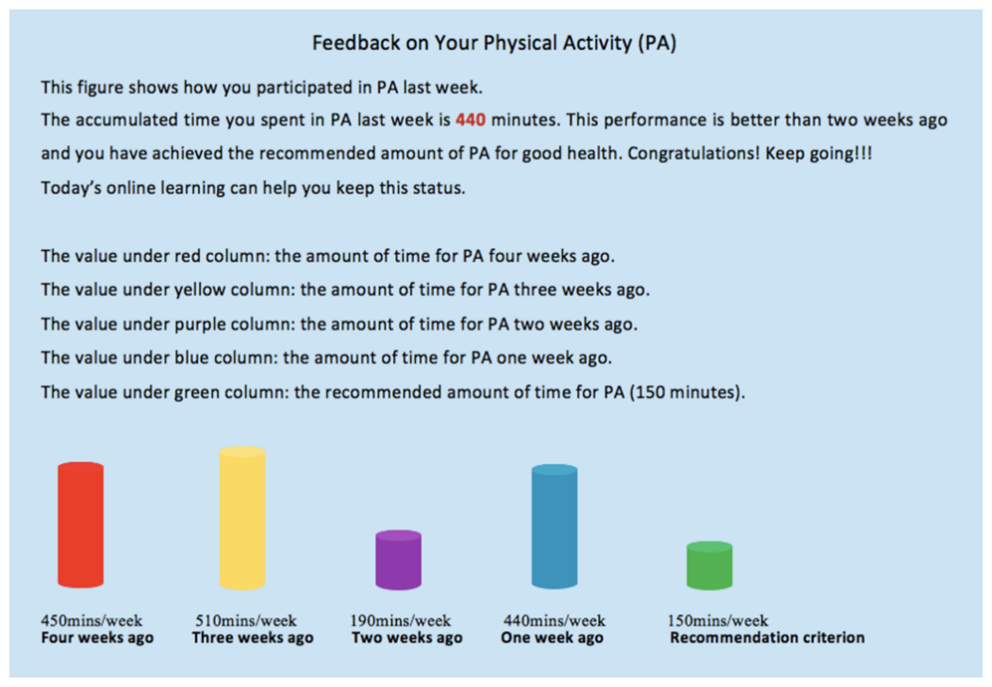
Example of individual and normative feedback relating to time spent engaging in physical activity.

The intervention was not tailored based on the motivational and volitional stage of the HAPA. Instead, participants received tailored individualized feedback on their behavior improvement or decline at the beginning of each session, based on their prior self-report questionnaire. Moreover, during each session, participants received tailored normative feedback in the form of bar charts to compare their current behavioral performance and progress with population recommendations. Figure 2 illustrates a screenshot example (translated from Chinese) of the feedback information the participants received for PA over an accumulated amount of time (in the previous 4, 3, and 2 weeks, and the first week), as well as the PA recommendation criteria. A tailored comment was also presented, such as

You have spent an accumulated 440 minutes in PA last week. Great! This performance is better than two weeks ago and you have achieved the recommended amount of PA for good health. Congratulations! Keep going!

In addition, we provided optional examples featuring role models throughout the intervention to support participants (eg, for setting goals or developing plans). We adopted a positive tone throughout the intervention.

The intervention in this study was based on previous research conducted in Germany and the Netherlands [23]. Prior to conducting the main study among Chinese university students, we completed preparation work on the intervention program in a prestudy, including the development and validation of adapted Chinese intervention materials, setting up intervention website modules, and optimizing website functioning by implementing Web-based tests with a small sample size.

### Measures

#### Demographic Information

Items addressed demographic characteristics, such as sex, age, and relationship status (single or in a relationship). We also collected self-reported body height (in centimeters) and body weight (in kilograms).

#### Health Behaviors

We assessed PA using the Chinese short version of the International Physical Activity Questionnaire (IPAQ-C) [24,25]. The IPAQ-C asked participants to estimate the number of days and amount of time spent on vigorous, moderate, and walking activities during the past 7 days. An individual total PA score (in minutes/week) was obtained when all questions were summed [25].

FVI during the past 7 days was assessed with 4 items: raw vegetables, fruits, fruit or vegetable juice, and cooked or steamed vegetables [26]. For each item, participants were asked to count the number of portions or glasses of liquid fruit and vegetables they consumed on average during a typical day. Each item had 11 options about the number of portions, such as 0, 0.5, 1, 1.5, 2, 2.5...5 or above. The total consumed portion was the sum of each item.

Stages of behavioral change were assessed for PA and FVI, each with 1 item on a 5-point scale asking “Did you engage in physical activity at least 5 days a week for 30 minutes or more (or 2.5 hours during the week)?” and “Please think about what you have typically consumed during the last weeks: did you eat 5 portions of fruit and vegetables per day?” (1=no, and I do not intend to start; 2=no, but I am considering it; 3=no, but I seriously intend to start; 4=yes, but only for a brief period of time; 5=yes, and for a long period of time) [27]. People who selected point 1 were nonintenders in stage 1, those who selected point 2 or point 3 were intenders in stage 2, while those who selected point 4 or point 5 were actors in stage 3.

#### Motivational Indicators of Behavior Change

We adapted the risk perception scale from Perloff and Fetzer [28]. Items started with the stem “How likely is it that you will have at some time in your life...,” followed by 5 items: “a high cholesterol level?,” “a heart attack?,” “high blood pressure?,” “a stroke?,” and “a cardiovascular disease?” Response used a 7-point scale ranging from 1=very unlikely to 7=very likely (Cronbach alpha=.84).

We assessed positive and negative outcome expectancies for PA with 2 items, each on a 5-point scale (1=don’t agree at all; 5=totally agree), such as “If I am physically active 5 days a week for 30 minutes or more, then...” “I feel better afterward,” or “it will cost me a lot of time” (positive: ρ=.78; negative: ρ=.57) [29]. We assessed positive and negative outcome expectancies for FVI, each with 2 items, such as “If I eat at least 5 portions of fruit and vegetables, then...” “this is good for my health,” or “this will be a financial burden” (positive: ρ=.88; negative: ρ=.74) [29].

We assessed self-efficacy for PA with 5 items on a 5-point scale (1=don’t agree at all; 5=totally agree), such as “I am certain that I can permanently be physically active for at least 5 days a week for 30 minutes each day” (Cronbach alpha=.88) [30]. We assessed self-efficacy for FVI by 5 items on the basis of the PA scale, such as “I am certain that I can eat 5 portions of fruit and vegetables a day even if it is sometimes difficult” (Cronbach alpha=.92) [30].

#### Volitional Indicators of Behavior Change

We distinguished planning as action planning and coping planning. We assessed action planning by the stem “For the next month I have carefully planned...” followed by 3 items for PA, such as “which PA I will pursue,” or followed by 3 items for FVI, such as “what I will eat” (Cronbach alpha for PA=.86; Cronbach alpha for FVI=.91). We assessed coping planning by the stem “For the next month I have carefully planned...” followed by 3 items for PA, such as “what I can do in difficult situations to stick to my intentions,” or followed by 3 items for FVI, such as “how I can eat healthy, even if something happened” (Cronbach alpha for PA=.87; Cronbach alpha for FVI=.93). Answers were given on a 5-point scale ranging from 1=totally disagree to 5=totally agree [15].

We assessed social support with 3 items for PA (Cronbach alpha=.72) [31] and with 3 items for FVI (Cronbach alpha=.69) [31], such as “My partner helps me/my family helps me/my classmates and friends help me to stay physically active,” or “My partner helps me/my family helps me/my classmates and friends help me to eat healthy.” Answers were measured with a 5-point scale (1=not at all true; 5=exactly true).

#### Distal Indicators of Behavior Change

Regarding intention, for PA, we assessed 3 independent items with a 4-point scale (1=not true; 4=exactly true), representing different intensity levels of PA. “On 5 days a week for 30 minutes (or a minimum of 2.5 hours per week), I have the intention to perform...” “strenuous physical activity,” “moderate physical activity,” or “mild physical activity” (Cronbach alpha=.34) [27]. We assessed intention regarding FVI using 3 items: “I seriously intend to...” “eat at least five portions of fruit and vegetables daily,” “eat fruit and vegetables at every meal,” and “drink at least one fruit or vegetable juice every day” (Cronbach alpha=.73) [27].

We assessed the habit scale with the stem “Being physically active for at least 30 minutes on 5 days a week is something that...” and “Eating 5 portions of fruit and vegetables per day is something that...” followed by 2 items, such as “has become a confirmed habit” and “I do without thinking about it” (Cronbach alpha for PA=.95; Cronbach alpha for FVI=.87). Answers were indicated on a 5-point scale ranging from 1=strongly disagree to 5=strongly agree [32].

#### Mental Health Outcomes

We assessed quality of life using the Hong Kong version of the World Health Organization’s Quality of Life-BREF questionnaire [33]. We measured general quality of life with the question “How would you rate your quality of life?” with a 5-point scale (1=very poor; 5=very good). We also used the physical health subdomain with 7 items (Cronbach alpha=.71), such as “How satisfied are you with your ability to perform your daily living activities?” (1=very dissatisfied; 5=very satisfied).

We assessed depression using the Chinese version of the Center for Epidemiologic Studies Depression (CES-D) scale, a self-report depression scale for research in the general population [34]. Participants were asked to indicate the frequency of symptoms on a 4-point scale (0=less than a day; 1=1 to 2 days; 2=3 to 4 days; 3=5 to 7 days) within the last week. Positively formulated items were reversed. The total score consisted of the sum of all 20 items and ranges from 0 to 60 (Cronbach alpha=.78). A CES-D score of 16 or greater indicated the likelihood of clinically significant depression.

Among all of the questionnaires above, IPAQ-C, quality of life, and depression had been well developed and validated in Chinese versions in previous studies [25,33,34]. The English questionnaires addressing motivational, volitional, and distal indicators of behavior change were translated into Chinese by a bilingual researcher. The translation was then validated using the standard back-translation technique [35]. Analysis of the data from the pilot test showed that the scales’ reliability was acceptable. In addition, we conducted pilot tests to ensure the usability and technical functionality of the electronic versions of the questionnaires prior to the main study.

### Data Analysis

We conducted all data analyses using IBM SPSS version 23 (IBM Corporation). We used independent samples *t* tests and chi-square tests to analyze dropout and to compare baseline characteristics at T1. Statistical significance was set at the 5% level (2-tailed).

We tested intervention effects on PA and FVI behavior (hypothesis 1a) with a repeated-measures multivariate analysis of variance after screening the variables successfully. To evaluate hypothesis 1b, we first presented descriptive information (count and percentage) on stage distribution across T1, T2, and T3 between the intervention group and the control group for PA and for FVI. We then performed chi-square tests. To increase the cell sizes of stage groups for statistical significance, we collapsed the nonintender and intender groups into an inactive group and contrasted it to the active group. We evaluated stage movements in terms of cross-tabulating stage at T1 with T2 or T3 separately for the intervention versus the control group to compare the 2 groups with each other.

In addition, we tested the effects on combined motivational, volitional, and distal indicators of behavior change (hypothesis 2), as well as on mental health outcomes (hypothesis 3), by conducting a series of repeated-measures analyses of covariance with baseline behavior as the covariate. For both repeated-measures multivariate analysis of variance and repeated-measures analyses of covariance, we tested trends over time (T1, T2, and T3) as the within-participants factor, with treatment (intervention group vs control group) as the between-participants factor, adjusting for both baseline PA and FVI (as covariates).

We report results based on those individuals who participated in all 3 measurement points. Missing data were imputed within each measurement point in time using the expectation-maximization method.

## Results

### Dropout Analysis

Results indicated that 493 people participated fully in data collection at T1, 337 at T1 and T2, and 142 at T1, T2, and T3. The dropout rates of participants were 31.6% (156/493) from T1 to T2 and 57.9% (195/337) from T2 to T3. The 142 final student sample included more women (n=101, 71.1%) than men, with a mean age of 19.3 years (range 17-24, SD 1.07). Most (128/142, 90.1%) of the participants were single. The average body mass index (BMI) of participants was 20.13 (SD 2.29) kg/m^2^. Participants at T1 and T2 (n=337) did not significantly differ from dropouts at T2 (n=156) with regard to relationship status (χ^2^_1_=0.06, *P*=.81), age (*t*_491_=–0.05, *P*=.96), BMI (*t*_491_=0.22, *P*=.96), amount of PA at baseline (*t*_491_=1.20, *P*=.23), and FVI at baseline (*t*_491_=–.87, *P*=.39). More women than men participated fully in data collection at T2 (χ^2^_1_=10.67, *P*=.001). In addition, those at T1, T2, and T3 (n=142) did not differ from dropouts at T2 and T3 (n=351) with regard to relationship status (χ^2^_1_=0.46, *P*=.49), age (*t*_491_=–1.46, *P*=.15), BMI (*t*_491_=1.12, *P*=.26), and FVI at baseline (*t*_491_=–0.74, *P*=.46). Those who participated in data collection at all 3 measurement points included more women than men (χ^2^_1_=22.60, *P*<.001) and engaged in less PA at baseline (*t*_491_=2.26, *P*=.02).

### Randomization Check

There were no differences across the 2 groups at T1 regarding PA level, FVI, social-cognitive indicators (risk perception, outcome expectancies, self-efficacy, action plans, coping plans, social support, intention, and habit), relationship status, and BMI (all *P* values .23-.94). Sex and age did, however, differ between the 2 groups, with more women (χ^2^_1_=7.650, *P*=.01) and younger participants (*t*_140_=–3.96, *P*<.001) in the intervention group than in the control group.

### Evaluation of Time and Treatment on PA, FVI, and Motivational, Volitional, and Distal indicators of Behavior Change

**Figure 3:**
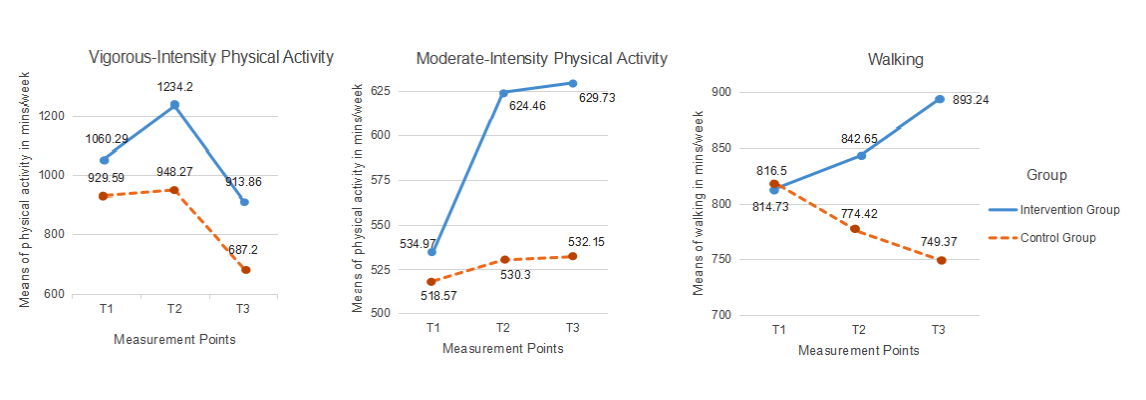
Performed physical activity (vigorous: left panel; moderate: middle panel; and walking: right panel) of the intervention group and the control group, in minutes per week, at 3 measurement points (T1: baseline; T2: end of intervention; and T3: 1-month follow-up).

**Figure 4:**
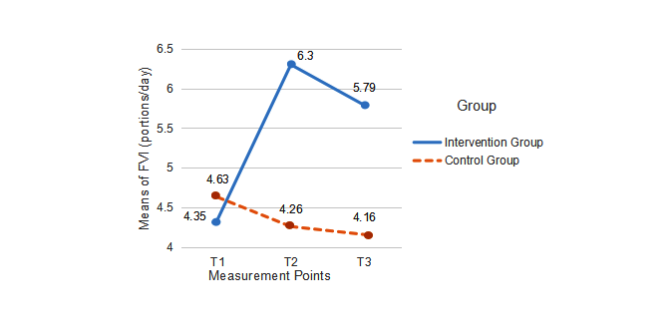
Fruit and vegetable intake (FVI) at T1 (baseline), T2 (end of intervention), and T3 (1-month follow-up) in the intervention group and the control group (portions per day).

Table 2 presents the results, most of which were significant. Because the question of whether the intervention group and the control group developed differently over time is crucial, the interaction was of the highest interest. Out of 8 effects, 7 were statistically significant, with an effect size of η^2^ ranging from .08 to .20. Only PA behavior was not statistically significant (see Table 2). However, the mean values of PA at different intensities (see Figure 3) indicated that there were descriptive differences between the 2 groups, which were clearly in favor of the intervention group. This was matched by the effects on FVI (see Figure 4), however, with significant effects (η^2^=.13, *P*<.001).

**Table 2:** Effect sizes (η^2^) and *P* values (significant at the 5% level, 2-tailed) for time, treatment group, and baseline behavior, as well as interaction.

Effects		Behavior^a^		Motivational^b^		Volitional^c^		Distal^d^	
		η^2^	*P* value	η^2^	*P* value	η^2^	*P* value	η^2^	*P* value
**Physical activity (minutes/week)**									
	Time	.03	.03	.04	.49	.07	.12	.04	.22
	Treatment	.01	.63	.05	.06	.11	<.001	.08	.01
	Baseline behavior	N/A^e^	N/A	.14	<.001	.10	.04	.19	<.001
	Interaction time ⨯ treatment	.01	.95	.11	.01	.14	.01	.08	.02
**Fruit and vegetable intake (portions/day)**									
	Time	.03	.15	.04	.52	.02	.81	.04	.22
	Treatment	.06	.01	.06	.05	.05	.06	08	.01
	Baseline behavior	N/A	N/A	.18	<.001	.13	<.001	.21	<.001
	Interaction time ⨯ treatment	.13	<.001	.14	.01	.20	<.001	.11	.01

^a^Behavior indicators: vigorous, moderate, and walking activity, or fruit and vegetable intake.

^b^Motivational indicators: risk perception, outcome expectancies, self-efficacy.

^c^Volitional indicators: action plans, coping plans, social support.

^d^Distal indicators: intention, habit.

^e^N/A: not applicable.

### Evaluation of Intervention Effects on Stage Progression to the Action Stage

Table 3 and Table 4 present the stage distributions at T1, T2, and T3 for PA and FVI. Descriptively, the stage developments underline that the intervention group was more likely than the control group to move to the action stage. After we collapsed the nonintender and intender groups as a single inactive group, there were at least 5 individuals in each cell for statistical significance testing. The findings validated previous results: for PA, individuals who were inactive at T1 in the intervention group were more likely to move to the action stage at T2 (χ^2^_1_=18.57, *P*<.001). Although descriptively this was also the case at T3, this intervention effect was not significant (χ^2^_1_=0.91, *P*=.34). While the overall intervention effect for individuals being inactive and active at T1 was also significant from T1 to T2 (χ^2^_1_=11.75, *P*=.001), no changes from T1 to T3 were statistically significant (all χ^2^ were <2 and *P* values ranged from .12 to .27).

**Table 3:** Physical activity stage distributions at T1, T2, and T3 (n=142).

Stage		Intervention group		Control group		Total	
		n	% within group	n	% within group	n	% within group
**At T1**							
	Nonintender	5	5.7	6	11.1	11	7.7
	Intender	49	55.7	25	46.3	74	52.1
	Actor	34	38.6	23	42.6	57	40.1
**At T2**							
	Nonintender	2	2.3	8	14.8	10	7.0
	Intender	30	34.1	23	42.6	53	37.3
	Actor	56	63.6	23	42.6	79	55.6
**At T3**							
	Nonintender	1	1.1	10	18.5	11	7.7
	Intender	42	47.7	20	37.0	62	43.7
	Actor	45	51.1	24	44.4	69	48.6

**Table 4:** Fruit and vegetable intake stage distributions at T1, T2, and T3 (n=142).

Stage		Intervention group		Control group		Total	
		n	% within group	n	% within group	n	% within group
**At T1**							
	Nonintender	7	8.0	6	11.1	13	9.2
	Intender	57	64.8	29	53.7	86	60.6
	Actor	24	27.3	19	35.2	43	30.3
**At T2**							
	Nonintender	1	1.1	8	14.8	9	6.3
	Intender	34	38.6	23	42.6	57	40.1
	Actor	53	60.2	23	42.6	76	53.5
**At T3**							
	Nonintender	1	1.1	6	11.1	7	4.9
	Intender	33	37.5	30	55.6	63	44.4
	Actor	54	61.4	18	33.3	72	50.7

Conducting the same tests for FVI, the findings revealed more positive results, with significant changes between the intervention and the control group, both at T2 for previously inactive (χ^2^_1_=15.07, *P*<.001) and active individuals (χ^2^_1_=4.28, *P*=.04), and for the overall effect (χ^2^_1_=15.64, *P*=.03). At T3, the effect was only significant in previously inactive individuals (χ^2^_1_=13.15, *P*<.001), but not active ones (χ^2^_1_=1.35, *P*=.21). The overall effect, however, was also significant (χ^2^_1_=10.52, *P*<.001), suggesting the intervention’s effectiveness.

### Evaluation of Time and Treatment Effects on Mental Health Outcomes

**Figure 5:**
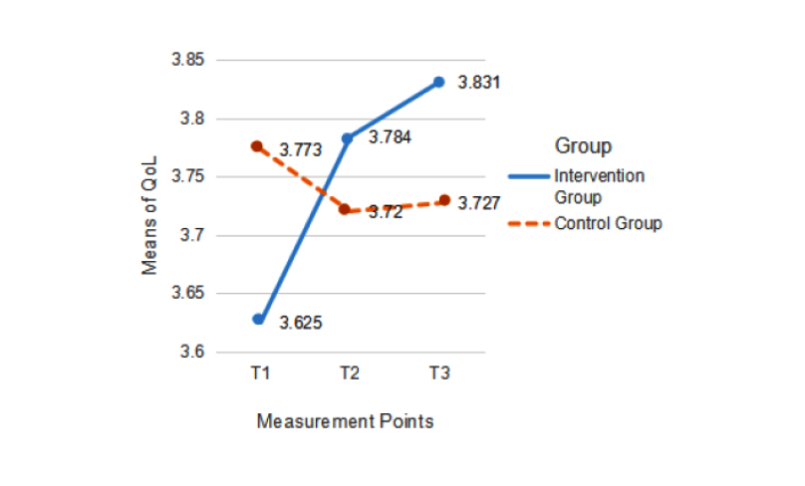
Mean scores for quality of life (QoL) at T1, T2, and T3 in the intervention group and the control group.

**Figure 6:**
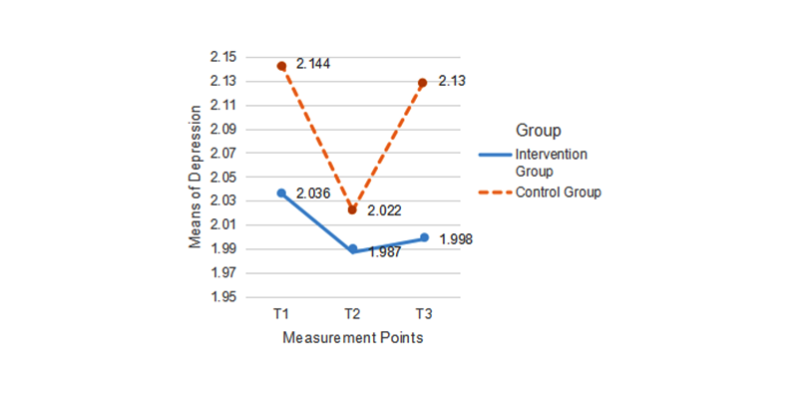
Mean scores for depression at T1, T2, and T3 in the intervention group and the control group.

Finally, we analyzed the intervention’s effect on quality of life and depression. As no significant differences between the stages emerged (tested for both behaviors), we left out the factor stage during the subsequent analysis. We found no significant differences for the group factor (*F*_3,139_=1.16, η^2^=.02, *P*=.31). The interaction of time and group was significant (*F*_3,139_=3.03, η^2^=.08, *P*=.02). The effects were, however, only due to quality of life (*F*_3,492_=1.23, η^2^=.03, *P*=.02). As for depression, the interaction was not significant (*F*_3,492_=0.17, η^2^<.01, *P*=.48). Figure 5 presents the mean values for quality of life and Figure 6 presents those for depression levels at the 3 measurement points for both groups.

## Discussion

This study aimed to test the efficacy of an 8-week Web-based intervention compared with a control group condition to improve PA and FVI in Chinese university students. The majority of the study assumptions were supported.

### Intervention Effects on Primary Outcomes

When testing the effects of time ⨯ treatment on the 2 behavior test variables, 1 was found to be significant. In comparison with the control group, students in the intervention group reported more consumption of fruit and vegetables over time. In addition, descriptively, the amounts of average FVI for the intervention group were all greater than the recommended amounts (5 portions per day) at the end of the 8-week intervention (6.3 portions) and at the 1-month follow-up (5.8 portions). This positive result among Chinese university students is consistent with a previous study, which was conducted with German and Dutch adults who wanted to reduce their cardiovascular risk [16,17]. Since we used intervention materials and a study design similar to the previous one, the generalization of intervention effects on dietary behavior in this study can be warranted across Eastern and Western countries.

In terms of PA behavior, however, there was no significant interaction effect, which is not in line with other studies [16,17,36,37]. Reasons for this discrepancy may include, first, that PA levels of university students were overall relatively high at the start of the intervention (T1). According to the IPAQ-C scoring protocol, more than half of the students in this study (268/493, 54.3%) were classified as “sufficiently active” at T1, which means that individuals already participated in 3 or more days of vigorous activity for at least 20 minutes per day [24]. Therefore, ceiling effects might have influenced the intervention’s efficacy on PA change. Second, as young adults need long-term processes to establish PA behaviors as habits, the 4-week Web-based intervention dose might not have been sufficient to change their PA habits. The same issue can also be found in another Web-based PA intervention among university students [38]. Third, measuring PA after 8 weeks might have been a too-distal measurement point, as PA was addressed only throughout the first 4 weeks. Hence, PA levels could have dropped back by the time of the assessment. Taking these findings together, half of hypothesis 1a was supported.

With respect to the intervention effects on stage progression for the two behaviors, the results were positive. In comparison with students in the control group, students who were inactive in the intervention group reported more stage movements to the action stage for PA from T1 to T2. In addition, the intervention effects on stage progression for FVI were found both from T1 to T2 and from T1 to T3. It seems that the stage progression of multiple health behaviors was positively interrelated with each other in this study (T1-T2). A previous study revealed that PA and FVI appeared to facilitate rather than hinder each other [39]. There were cross-behavior associations between these two behaviors. This opinion was also supported by a study that found consistently significant correlations across stages between nutrition and PA (*r* range .16-.26, *P*<0.01) [40]. Future studies should address the evaluation of mechanisms that transfer intervention effects on stage movement from PA to FVI, or vice versa. Referring back to hypothesis 1b, the data supported most of the assumption of stage progression.

### Intervention Effects on Secondary Outcomes

When evaluating the outcomes of social-cognitive indicators of behavior change, all 6 tests revealed significant treatment effects on motivational, volitional, and distal indicators of PA and FVI over time, with the effect size ranging from .08 to .20. The findings are in line with previous studies, in which motivational and volitional interventions were both used to change multiple health behaviors [8,16,17]. In sum, our results support hypothesis 2.

The increase in positive mental health outcomes of quality of life and depression levels was evident in this study when combining the 2 measures together. This main effect, however, was due to the changes in quality of life only and not significantly to changes in depression. One possible reason for the lack of effect on depression might be that PA did not improve in this study, which cannot bring about mental health consequences. Another possible cause might be floor effects, as university students reported low depression levels at the start of the study (mean 2.08, SD 0.61), which reflects the healthy mental status of participants in this study. To prevent depression in the long term, however, more components explicitly addressing mental health would need to be developed and tested in future interventions. Overall, half of hypothesis 3 was supported.

### Limitations

Some limitations of this study need to be addressed. First, the dropout rate was high. Compared with male students, female students who performed less PA at baseline were more likely to comply with the instructions of the program and spent time completing data collection across the 3 time points. Thus, personal characteristics could be one explanation for the high dropout rate. Another reason might be related to the physical education lecturers’ verbal reminders in physical education class. In this study, data collection at T3 was scheduled during the week in which the last physical education class was offered. Students who attended the final physical education examination 1 week before the last class were allowed to be absent for the last physical education class, which means the lecturers could not verbally encourage those absent students to complete the final data collection. This might explain why the dropout rate from T2 to T3 (57.8%) was higher than that from T1 to T2 (31.6%). In addition, dropout and low engagement with questions could be caused by intervention features, such as the Web-based format and layout, length of the questionnaire, or browser difficulties on the intervention website [41-43]. Future studies should further address the characteristics of dropout and nonresponse to Web-based interventions, and find a solution to enlarge the sample size. This would also help provide more comprehensive subgroup analyses on the effectiveness of specific tailored components, as all participants would receive a unique intervention, but are treated equally when analyzing the data [44].

Second, the use of self-report questionnaires for behavioral outcome measures could have led to recall bias, overreporting or underreporting, and measurement errors. Thus, the inclusion of objective measures such as biomarkers, accelerometers, or pedometers is desirable in future studies. Third, the follow-up period was relatively short in this study. The longer-term impact of the intervention should be assessed in the future. Fourth, this study did not explore the mechanism of how the treatment facilitated multiple health behavior change (ie, the synthesis of PA and FVI). In other words, it is unclear which social-cognitive factors mediated the effect of the intervention on the improvements in health behavior. We advocate conducting an in-depth test in future studies.

### Conclusions

This study provides evidence for the efficacy of a Web-based multiple health behavior intervention among Chinese university students. The majority of study hypotheses were supported. The initial findings suggest that the intervention was effective at increasing FVI and in enhancing perceived quality of life. In addition, all social-cognitive indicators of PA and FVI were improved in this study. The intervention did not, however, show the hypothesized effect on PA change. Future research is warranted to address some of the limitations noted above, especially reducing high dropout rates and exploring the most effective components of the intervention, which is imperative to facilitate health promotion among university students, not only in the Western but also in the Eastern hemisphere.

## Appendix

Multimedia Appendix 1 CONSORT-EHEALTH form V1.6.

## Abbreviations

BMI: body mass index
CES-D: Center for Epidemiologic Studies Depression
FVI: fruit and vegetable intake
HAPA: health action process approach
IPAQ-C: International Physical Activity Questionnaire Chinese short version
PA: physical activity
